# BAF45D-binding to *HOX* genes was differentially targeted in H9-derived spinal cord neural stem cells

**DOI:** 10.1038/s41598-023-50939-y

**Published:** 2024-01-02

**Authors:** Chang Liu, Yuxin Xie, Xueying Chen, Lihua Liu, Chao Liu, Zongsheng Yin

**Affiliations:** 1 Department of Orthopedics, The First Affiliated Hospital, Anhui Medical University, Hefei, 230032 Anhui China; 2 Department of Histology and Embryology, Institute of Stem Cell and Tissue Engineering, School of Basic Medical Sciences, Anhui Medical University, Hefei, 230032 Anhui China; 3 Institute of Clinical Pharmacology, Anhui Medical University, Hefei, 230032 Anhui China

**Keywords:** Computational biology and bioinformatics, Neuroscience, Stem cells

## Abstract

Chromatin accessibility has been used to define how cells adopt region-specific neural fates. BAF45D is one of the subunits of a specialised chromatin remodelling BAF complex. It has been reported that BAF45D is expressed in spinal cord neural stem cells (NSCs) and regulates their fate specification. Within the developing vertebrate spinal cord, *HOX* genes exhibit spatially restricted expression patterns. However, the chromatin accessibility of BAF45D binding *HOX* genes in spinal cord NSCs is unclear. In the present study, we found that in H9-derived spinal cord NSCs, BAF45D targets *TBX6*, a gene that regulates spinal cord neural mesodermal progenitors. Furthermore, BAF45D binding to the *NES* gene is much more enriched in H9-derived spinal cord NSCs chromatin compared to ESCs chromatin. In addition, BAF45D binding to anterior and trunk/central *HOX* genes, but not to lumbosacral *HOX* genes, was much more enriched in NSCs chromatin compared to ESCs chromatin. These results may shed new light on the role of BAF45D in regulating region-specific spinal cord NSCs by targeting *HOX* genes.

## Introduction

Human embryonic stem cells (ESCs) can be efficiently induced into spinal neural cells with discrete *HOX* gene profiles by temporal regulation of Wnt, FGF and retinoic acid (RA) signalling^1^. The induction of spinal cord HOX genes corresponds to a region-wide loss of inhibitory chromatin marks that regularly occur in more anterior neural precursors^2^. The molecular mechanism underlying this response is closely linked to chromatin remodelling in ESCs^3^.

*HOX* gene expression functions in the development of the rhombomere hindbrain (*HOX1–5*), cervical (*HOX5–9*), thoracic (*HOX9–10*) and lumbosacral (*HOX10–13*) segments of the vertebral spinal cord^4^. In vertebrates, the *HOX* gene is organised into homologous groups (HG) 1–13, which exist on multiple chromosomes due to genomic replication events^5^. In mammals, four different chromosomes carry the *HOX* gene, known as the *HOX* A-D cluster. *HOX* genes are usually divided into anterior (PG1–3/4), main/central (PG4/5–9) and posterior (PG10–13) variants, with the order of each cluster reported^6^.

Chromatin accessibility defines how cells adopt region-specific neural fates^7^. During an in vitro motor neuron differentiation system that recapitulates embryonic development, the genomic binding profiles of different *HOX* genes are diverse, driving the diversification of neural patterning^8^. The BRG1/BRM-associated factor (BAF) complex, the mammalian chromatin remodeller, modulates chromatin accessibility to activate and/or repress target genes^9,10^. For example, rapid BAF depletion has been reported to redistribute chromatin regulators, such as the Polycomb Repressive Complexes (PRCs), from highly occupied *Hox* genes to weakly occupied genes, which are typically counteracted by the BAF complex^11^. In human pluripotent stem cell (hPSC)-derived neuromesoderm, which exhibits colinear *HOX* gene expression, retinoic acid silences expression and transforms the neuromesoderm into neuroectoderm with distinct *HOX* gene/protein profiles that can be additionally induced into site-specific neural cells^4^.

BAF complexes consist of three subunits, the ATPase, the actin-related protein (ARP) and the core subunit^12^. Among the BAF complex subunits, BAF45D (DPF2) belongs to the core subunits^12–14^. Recently, BAF45D is expressed in spinal cord neural stem cells (NSCs) and is required for the expression of PAX6, a neuroectodermal marker, during RA-induced H9 cell differentiation^15^. In addition, BAF45D regulates the fate of spinal cord NSCs through the SMAD-PAX6 axis^16^, although the role of BAF45D in anterior–posterior *HOX* gene modulation is not clear.

Spinal cord NSCs have been identified in and outside the central canal and may serve as endogenous NSCs for regenerative therapy of SCI^17,18^. Human ESC-derived spinal cord NSCs are critical for in vitro evaluation of spinal cord development and cell replacement therapy in spinal cord disease. However, the role of chromatin accessibility of BAF45D-bound *HOX* genes in spinal cord NSCs is largely unknown. Here, we hypothesised that BAF45D may modulate *HOX* gene expression by regulating chromatin accessibility and used spinal cord NSCs derived from H9 cells to investigate how BAF45D targets *HOX* genes.

## Results

### Different BAF45D-binding gene profiles between undifferentiated H9 cells and spinal cord NSCs derived from H9 cells

**Figure 1:**
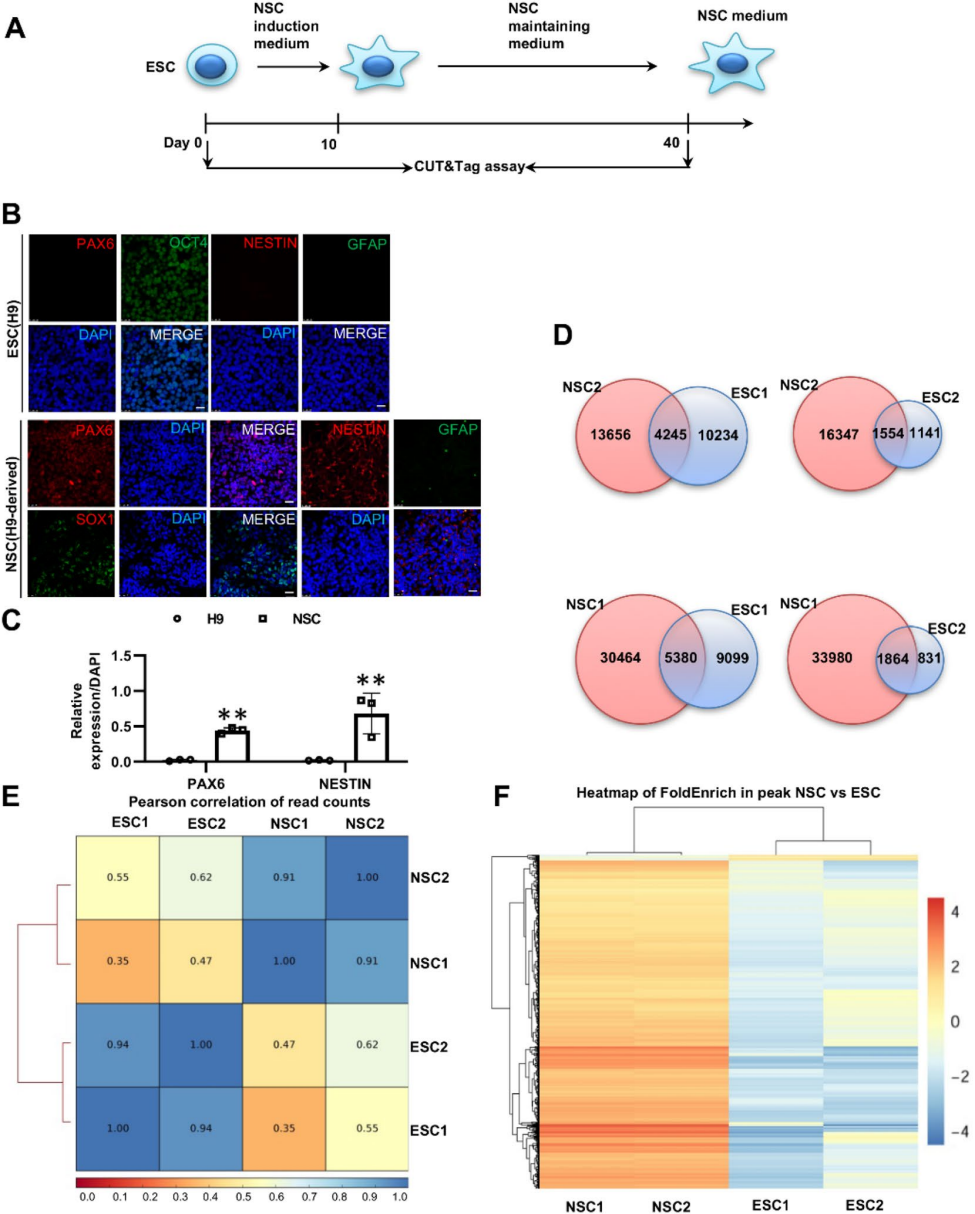
Chromatin accessibility of BAF45D binding gene profiles between H9 cells and H9-derived spinal cord NSCs. (**A**) Overview of the experimental procedure. Differentiation of H9 cells into spinal cord NSCs in response to NSC induction medium, NSC maintenance medium and NSC medium. The CUT&Tag assay was performed at the indicated time point. (**B**) IF assay for the expression of OCT4, PAX6, NESTIN, SOX1 and GFAP in H9 cells and H9-derived spinal cord NSCs. Scale bar = 25 μm. (**C**) Statistical analysis for relative expression of PAX6 and NESTIN. (**D**) Peak differences between ESC and NSC groups. The number of common and unique peaks in the comparison group is shown in the Venn diagram. (**E**) Pearson correlation coefficient between CUT&Tag biological replicates. (**F**) The cluster graph of peak correlation of different experimental groups in the comparison groups, showing a heat map of FoldEnrich in peaks. The heatmap was generated using R software (https://cran.r-project.org/) with a pheatmap package (1.0.12, https://cran.r-project.org/web/packages/pheatmap/index.html).

To investigate the different gene profiles between undifferentiated H9 cells and spinal cord NSCs derived from H9 cells, we induced H9 cells into spinal cord neural stem cells according to a previous protocol^16^ (Fig. 1A) using NSC induction medium, NSC maintenance medium and NSC medium. The IF assay results showed that while undifferentiated H9 cells did not express SOX1 and H9-derived spinal cord NSCs did not express OCT4 (data not shown), the expression of OCT4 in undifferentiated H9 cells and the expression of PAX6, SOX1, NESTIN and GFAP in H9-derived spinal cord NSCs can be identified (Fig. 1B). Statistical analysis to quantify the expression of PAX6 and NESTIN indicated a significant increase in both marker proteins (Fig. 1C). BAF45D has been reported to be expressed in both undifferentiated and differentiated H9 cells^15^ . HOXC9 is known as a thoracic/central spinal cord marker protein^19–21^. To investigate the expression of BAF45D and HOXC9 in H9-derived spinal cord NSCs, we performed an IF assay and showed colocalisation of BAF45D and HOXC9 in the spinal cord NSCs (Fig. S1A–U).

Previously, we reported the genomic distribution of binding sites in relation to the distance to the TSS of target genes and in relation to genomic elements (intergenic, exon, intron, promoter)^16^. Here, we used the data sets and performed new analysis to show the peak differences between the H9 and NSC groups and the common and unique number of peaks in the comparison groups are shown in the Venn diagram (Fig. 1D), indicating the different gene profiles. Next, the biological replicates of the CUT&Tag assay were monitored by the Pearson correlation coefficient (Fig. 1E). In addition, the cluster graph of the FoldEnrich heatmap in the peak correlation of the different experimental groups also showed the different gene profiles (Fig. 1F). Moreover, the analysis to find enriched DNA binding motifs was performed across all BAF45D bound regions in the H9 cells and H9-derived spinal cord NSCs. DNA motifs of *HOXC9*, *HOXA9* and Regulatory factor X (RFX) (*RFX2* and *RFX3*) were found to be enriched in the bound regions in the two NSC samples (Figure S2). The enrichment of the *HOXC9* motif is in line with our expectations, whereas the enrichment of *HOXA9* (data not shown in Figure S1) comes as a surprise to us. In addition, we found that the top two homer de novo motifs for BAF45D include *HOXC9* and *RFX1* (Figure S3). As a family of transcription factor, RFXs regulate the maintenance of NSCs^22^**.** Gene ontology (GO) analysis of enriched bound genes (Fig. S4) related to neural tube development also indicated that the neural tube genes are enriched in the NSC samples.

These results suggest that BAF45D bound gene profiles are much more enriched in H9-derived spinal cord NSCs compared to ESCs.

### BAF45D targets *TBX6* but not *OTX2* in human spinal cord NSCs

**Figure 2:**
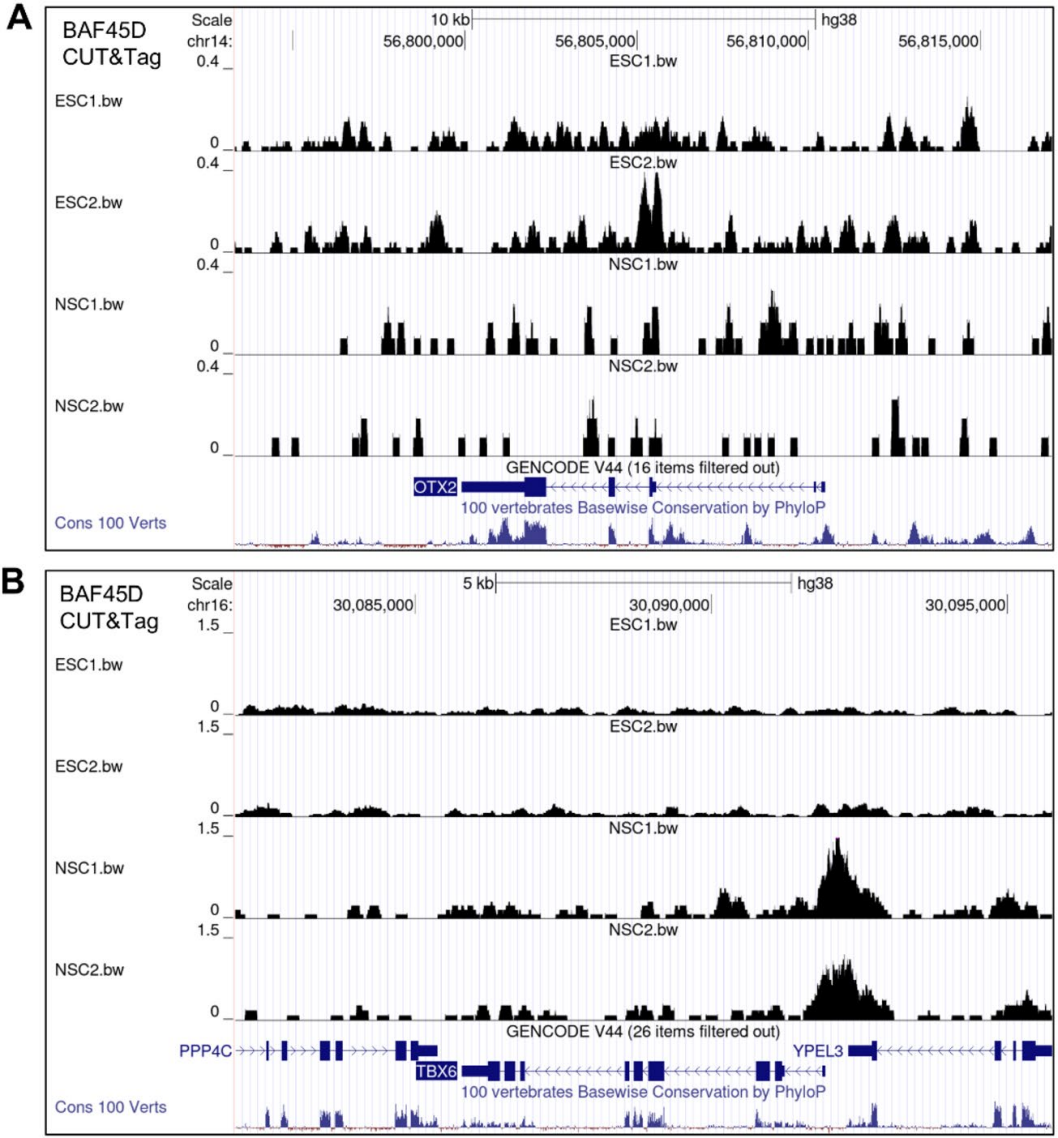
BAF45D targets genes that regulate neural mesodermal progenitors in the spinal cord. (**A**) Chromatin accessibility of BAF45D-bound *OTX2*, a brain marker, was not enriched in NSCs compared to H9 cells. (**B**) Chromatin accessibility of BAF45D-bound *TBX6*, a spinal cord neural mesodermal progenitor marker, in NSCs was enriched compared to that in H9 cells. Visualization of the sequencing data together with the conservation data by UCSC genome browser was shown.

Neural mesodermal progenitors (NMPs) contribute to both spinal cord and mesodermal cells^23^. *Tbx6* is expressed in a novel transient NMPs^24^, and NMPs differentiate into paraxial mesodermal cells that begin to express *TBX6*^25^, suggesting the possibility that *TBX6* is a potential marker for both NMPs and mesodermal cells. To further identify the different gene profiles between H9 cells and H9-derived NSCs, we examined the chromatin accessibility of BAF45D-bound *OTX2*, a brain marker, and *TBX6* in both human NSCs and H9 cells. The results showed that the chromatin accessibility of BAF45D-bound *OTX2* (Fig. 2A) was not increased in human NSCs, whereas the chromatin accessibility of BAF45D-bound *TBX6* (Fig. 2B) was increased in NSCs compared to H9 cells. To exclude the experimental bias of the analysis, visualization of two less enriched BAF54D bound genes, *POU5F1 (gene of OCT4)* and *HOXC13*, were shown (Figure S5.). There are inverse data showing that BAF45D binding to *POU5F1 and HOXC13* is lower in the NSC group than that in the H9 cell group.

These results suggest that BAF45D preferentially regulates genes in the spinal cord.

### BAF45D binds more *NES *and the anterior *HOXB1* gene in human spinal cord NSCs

**Figure 3:**
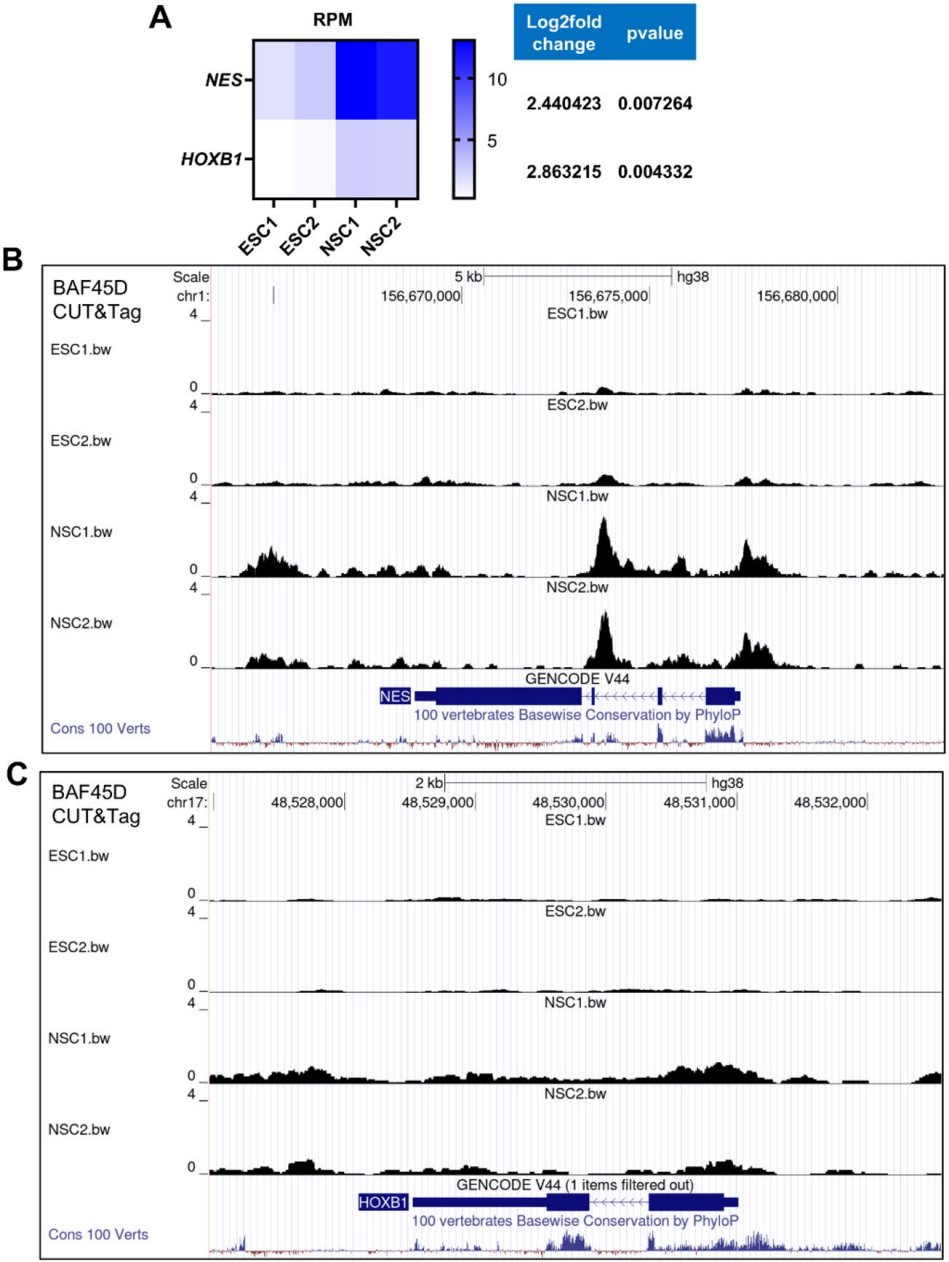
BAF45D targets more *NES* and *HOXB1*. (**A**) RPM values of peaks associated with BAF45D-bound *NES* and *HOXB1*. (**B, C**) Visualization of the custom sequencing data together with the conservation data by UCSC genome browser for the indicated sequences of the *NES* (**B**) and *HOXB1* (**C**) genes.

Next, we wanted to investigate changes in chromatin accessibility of BAF45D with the NSC marker and anterior *HOX* genes. The RPM (the proportion of reads enriched in the peak region per million reads) values of each sample at the peak sites were calculated. The RPM values at the sites of the NSC marker gene *NES* and the *HOX* gene *HOXB1* in the H9 cells were increased compared to those in the H9-derived NSCs (Fig. 3A). Furthermore, visualization of the CUT&Tag tracks at the *NES* and *HOXB1* loci (Fig. 3B, C) indicated the chromatin accessibility of BAF45D was increased. Previously, we reported that knockdown of Baf45d (also called Dpf2) decreased *Hoxb1* during RA-induced neural differentiation of P19 cells, supporting that the BAF45D binding to *Hoxb1* correlates with direct target gene activation^16^.

These results suggest that chromatin accessibility plays a critical role in NSC induction in the spinal cord.

### *BAF45D/DPF2* bound histone marks are functionally relevant

**Figure 4:**
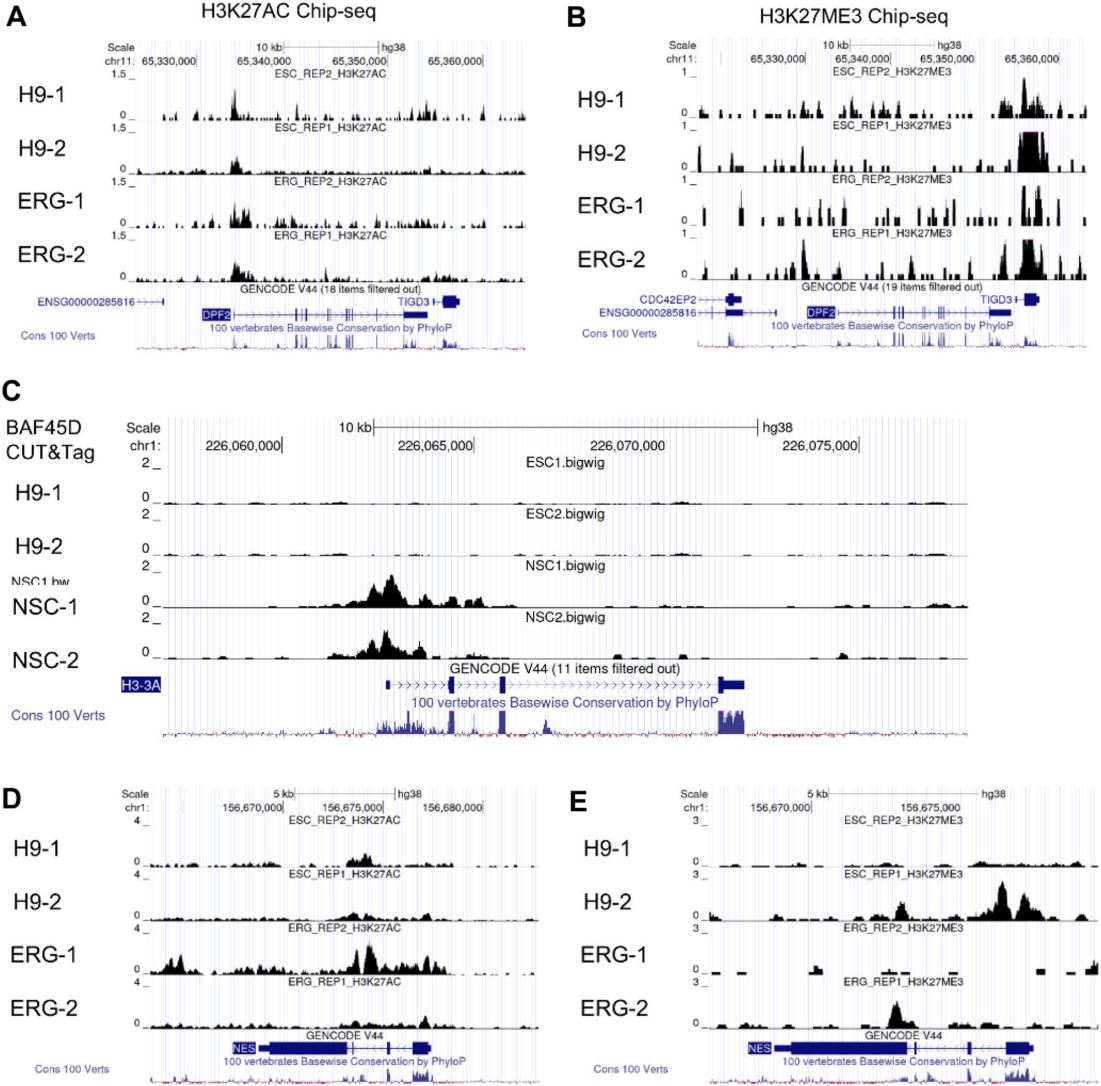
Function of *BAF45D/DPF2* bound histone marks. (**A**) Visualisation of H3K27ME3 enriched chromatin was plotted over the DPF2 locus in H9 cells and H9-derived early radial glial (ERG) cells. (**B**) Visualisation of H3K27AC enriched chromatin was plotted over the DPF2 locus in H9 cells and H9-derived early radial glial (ERG) cells. (**C**) The binding profile of BAF45D within the H3 locus. (**D,E**) The data of H3K27ME3/H3K27AC binding to NES are shown.

To further address the BAF45D bound regions are functionally relevant, we used publicly available Chip-seq data sets (http://cistrome.org/db/#/) of both H9 cells and H9-derived neural progenitor cells published in a previous paper ^26^. The data sets were analysed using the UCSC genome browser linked to the website. It has been reported that radial glial cells (RGCs) are derived from neuroepithelial (NE) cells ^27^and can function as NSCs^28^. Furthermore, early RGCs(ERGs) serve as neural progenitor cells(NPCs)^29^, so we selected the ERG stage to mimic our H9-differentiated NSCs The levels of H3K27ME3 and H3K27AC were plotted across the BAF45D/*DPF2* locus. Compared to the levels for H3K27ME3 binding to BAF45D/*DPF2*, the levels for H3K27AC binding to BAF45D/*DPF2* were much more enriched in both H9 cells and the ERGs. Furthermore, the peak enrichment of H3K27ME3 binding to BAF45D/*DPF2* was different from that of H3K27AC binding to BAF45D/*DPF2* (Fig. 4A, B).

We also showed the binding profile of BAF45D within the *H3* locus to demonstrate the BAF45D-*H3* correlation (Fig. 4C) and the data of H3K27ME3/H3K27AC-binding to *NES* (Fig. 4D, E). In addition, the ChIP-seq enrichment profiles of H3K27ME3 and H3K27AC histone marks in across genomic regions centered at BAF45D peak summits were shown (Fig. S6). Since H3K27ME3 may play a role in repressing loci and H3K27AC may play a role in activating enhancers and/or promoters in embryos^30^. These data suggest that BAF45D bound regions are functionally relevant.

### Chromatin accessibility of BAF45D binding anterior and trunk/central *HOX* genes was enriched in human spinal cord NSCs

**Figure 5:**
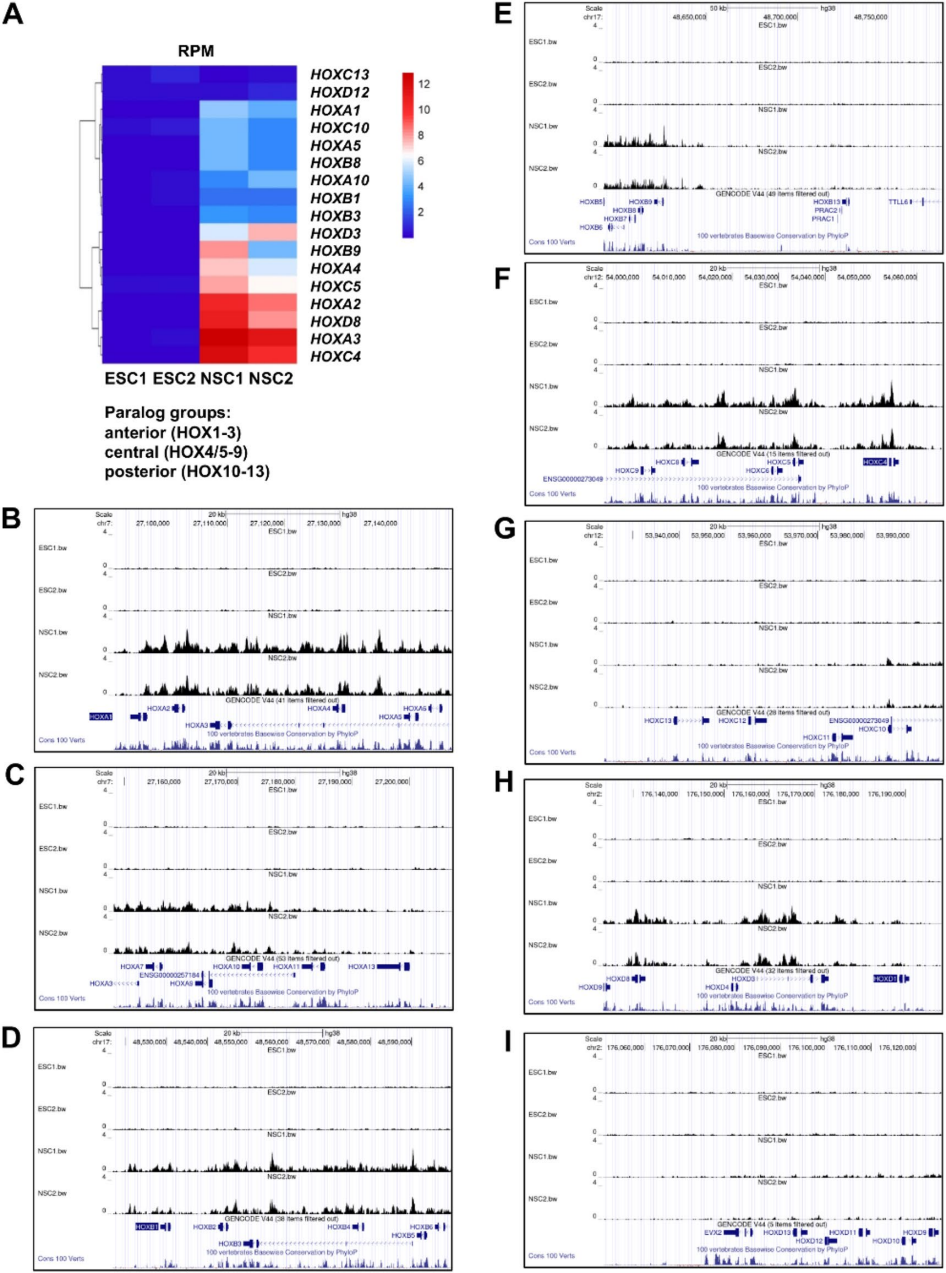
Chromatin accessibility of BAF45D binding anterior and trunk/central *HOX* genes was increased in H9-derived NSCs. (**A**) RPM values of peaks in different samples of *HOX* genes. (**B–I**) Visualization of the custom sequencing data together with the conservation data by UCSC genome browser for the anterior (PG1–3), trunk/central (PG4/5–9) and posterior (PG10–13) clusters of *HOXA* (**B,C**), *HOXB* (**D,E**), *HOXC* (**F,G**) and *HOXD* (**H,I**).

Within the developing spinal cord in vertebrate embryos, *HOX* genes display spatially restricted expression patterns and are thought to be critical for determining cell specification and segmental identity along the anterior–posterior axes of the spinal cord^5^. Early segmental expression patterns can regulate the formation of complex spinal cord circuits^31^. We examined the general chromatin accessibility of BAF45D binding to *HOX* genes. We found that among the BAF45D-binding *HOX* genes, the RPM values (Fig. 5A) of the *HOX* gene peaks indicated that the accessible chromatin of the anterior (PG1–3) and trunk/central (PG4/5–9) *HOX* genes appeared to be different from that of the posterior (PG10–13) *HOX* clusters. Visualization of BAF45D binding *HOX* genes further supported that chromatin accessibility of BAF45D-bound anterior and trunk/central, but not posterior (*HOX12–13*) genes was increased in H9-derived spinal cord NSCs (Fig. 5B–L). Further analysis showed that overexpression of GFP-tagged BAF45D, but not GFP, increased HOXC9 expression in the RA-treated P19 cells (Fig. S7). However, we did not find expression of HOXB13, a gene that control axial progenitor activity in the tail bud^32^, in the P19 cells (data not shown). In addition, H9-derived NSCs expressed robust HOXB1 but little or no HOXB13 (Fig. S6, I–M). These results may suggest that BAF45D is involved in the region-specific binding of *HOX* genes.

## Discussion

In this study we have made some novel findings: BAF45D targets *TBX6* but not *OTX2* in spinal cord NSCs derived from H9 cells; the chromatin accessibility of BAF45D-bound *NES* and *HOXB1* was increased in human spinal cord NSCs; and the chromatin accessibility of BAF45D-bound anterior and trunk/central but not posterior *HOX* genes was increased in human spinal cord NSCs.

The BAF complex plays a crucial role in the regulation of gene expression and neuronal differentiation. Cell-specific BAF function and developmental regulation starts with the combined arrangement of different BAF complexes, such as the neuronal BAF (nBAF) complex, the neural progenitor BAF (npBAF) complex and the embryonic stem cell-specific BAF (esBAF) complex. Therefore, understanding how the subunit of the BAF complex regulates its role and which function each subunit regulates will highlight the in vivo role of different subunits^14,33^.

Human pluripotent stem cells (hPSCs) treated with Wnt activator (CHIR-99021) and leukaemia inhibitory factor (LIF) in N2B27 medium were induced into NSCs, with the expression of some *HOX* genes, including *HOXB1*^34^. Here, we generated NSCs from H9 cells using N2B27 medium supplemented with CHIR-99021, LDN-193189, SB431542, FGF2, FGF8 and DAPT. The H9-derived NSCs are characterised by the expression of NESTIN and HOXC9, indicating a spinal cord characterisation. In addition, BAF45D targets *TBX6*^35^, a neural mesodermal progenitor marker, more strongly in H9-derived NSCs than in H9 cells. Neural mesodermal progenitors (NMPs) are known to be involved in spinal cord development during axial elongation^36^, supporting the idea that BAF45D may promote the induction of human spinal cord NSCs. In addition, chromatin accessibility of BAF45D-bound *HOXB1* was enriched in H9-derived spinal cord NSCs. H9-derived spinal cord NSCs expressed robust HOXB1 but little or no HOXB13. Given that BAF45D determined spinal cord NSC induction and BAF45D knockdown decreased Hoxb1 expression^16^, these data suggest that BAF45D may differentially target *HOX* genes and that *HOXB1* may be a target of BAF45D. Because Hoxb1 determines cell fate and promotes the proliferation of NSCs^37^,we therefore speculate that BAF45D may contribute to the proliferation of human spinal cord NSCs.

In the Venn diagram, there is a discrepancy in the total number of peaks between ESC1 (~ 14k peaks) vs ESC2 (~ 2.6k peaks). However, there is also a discrepancy between NSC1 (~ 35k peaks) vs. NSC2 (~ 17k peaks). We speculate that this may be due to the different initial states of the two replicates. As this study mainly focuses on the changes in BAF45D binding to gene profiles between the undifferentiated H9 cells and the differentiated H9-derived NSCs. The discrepancies at both the start and end point may also indicate the similar changes between the ESCs and NSCs. In addition, BAF45D binding is much more enriched in NSCs chromatin compared to ESCs chromatin and there is only a very small fraction of peaks that are enriched in ESCs chromatin. We have reported that BAF45D/DPF2 targets OCT4, a stem cell marker protein, and contributes to the differentiation of H9 cells^15^. Since the spinal cord genes are enriched in the NSC samples (Figure S4), the much more enriched BAF45D binding in NSCs may indicate that BAF45D targets more genes, including neural tube development genes.

Finally, BAF45D-binding *HOX* genes, were enriched in human spinal cord NSCs. In a published study, activation of *Hox* genes was found after three days of conditional knockout of Brg1^38^. During the formation of the posterior central nervous system (CNS), *HOX* gene expression was generated within the neuroepithelium of the hindbrain (*HOX1–5*) and spinal cord cervical (*HOX5–9*), thoracic (*HOX9–10*) and lumbosacral (*HOX10–13*) segments. The spatial variation of *HOX* gene expression along the rostrocaudal (R/C) axis precisely directs the development of different neural subtypes to discrete axial positions and induces different lineages of neuroepithelial progeny^39^. Indeed, a previous publication reported that during hPSC neural differentiation, much more time was required for the expression of lumbosacral *HOX* genes compared to cervical and thoracic *HOX* genes^4^. This report may provide an explanation for our finding that BAF45D binds more anterior and thoracic/central *HOX* genes but not posterior *HOX* genes in H9-derived spinal cord NSCs. According to our CUT&TAG assay data, the visualization results obtained by UCSC genome browser showed that BAF45D binding to *HOXB1* and *HOXC9* genes was enriched in the H9-derived NSCs.

Here BAF45D protein colocalised with HOXC9 protein in most of the NSCs. We hypothesised that HOXC9 may act as a co-regulator for spinal cord neural cell fate decision. Hoxc9 activity is essential for neuron subtype identity at the thoracic level ^20^, and is expressed by motor neurons and interneuron progenitors at the thoracic level in vivo ^40^. BAF45D is also expressed in spinal cord NSCs and neurons in vivo^41^. Therefore, our in vitro data together with the in vivo settings support the hypothesis that BAF45D may cooperate with HOXC9 and regulate thoracic NSC fate decision.

We also found enriched motifs for *HOXC9* and *RFXs* in BAF45D-bound regions. RFX1 belongs to RFX transcription factors that regulate the maintenance of neural stem cells^22^. Interestingly, *RFX2* and *RFX3* motifs were also found in the top three Homer known motifs for BAF45D. These findings may further support the potential role of BAF45D in human spinal NSC maintenance.

It is known that CUT&Tag assay provides efficient high resolution sequencing libraries for profiling diverse chromatin features. Mapping of specific chromatin features in cells may help researchers to understand how genes were regulated, such as histone modifications ^42^. We demonstrate that BAF45D bound regions are functionally relevant to two histone marks, H3K27ME3 and H3K27AC, which regulate gene repression and activation, respectively. It is currently unclear how the expression profile of BAF45D and *HOX* genes correlates in vivo. The BAF complex is known to be critical for Polycomb-mediated repression in ESCs during lineage commitment characterised by *Hox* gene disinhibition^38,43^. Brg1 is essential for regulating active and repressive chromatin states during mesoderm lineage specification^43^. In particular, Brg1 degradation induces derepression of *Hox* genes along with upregulated genes associated with neural tube development^11^. Since BAF45D is a subunit of the polymorphic canonical BRG1-associated factor (cBAF) complex, we speculate that BAF45D may play a role in *HOX* gene expression in vivo. However, the effect of BAF45D on *HOX* gene expression in spinal cord NSCs is still unclear and deserves to be investigated in the near future.

Taken together, our findings suggest that BAF45D may target different *HOX* genes and contribute to the fate decisions of human spinal cord NSCs.

## Methods

### H9 cell culture

A human embryonic stem cell line, H9 cells (ordered from Guangzhou Guiyi Biotechnology Co. Ltd.), was routinely maintained according to previous protocols^15,16^. Briefly, a six-well plate was precoated with Matrigel solution (Corning, 356234). H9 cells were thawed and resuspended in mTesRTM1 medium (Stem Cell Technologies, 85850). The suspended cells were then replated onto Matrigel coated culture plates and cultured at 37 °C in 5% CO_2_. When the H9 cells reached 80% confluence, single cell passaging was performed. For single cell passaging, H9 cells were treated with mTeSRTM1 medium supplemented with 10 μM Y27623 for 1 h and exposed to Accutase (1 mL/well) at 37 °C. The cells were collected in a 15 mL centrifuge tube. After centrifugation at 500 rpm for 5 min, the cells were resuspended in mTesRTM1 medium supplemented with 10 μM Y27623 and seeded into six-well plates coated with Matrigel. The use of H9 cells was approved by the Biomedical Ethics Committee of Anhui Medical University. All experiments were performed according to the relevant guidelines and regulations.

### Induction of spinal cord neural stem cells from H9 cells

Induction of spinal cord neural stem cells from H9 cells was performed according to a previously described protocol^1^. Briefly, H9 cells were passaged in the single cell mode. When the cells reached 70% confluence, the mTeSRTM1 medium was changed to the NSC induction medium (N2B27 medium supplemented with 10 μM DAPT, 4 μM CHIR-99021, 10 μM SB431542, 100 nM LDN-193189, `100 ng/mL FGF2 and 100 ng/mL FGF8). When the cells reached more than 90% confluence, the cells were passaged at a ratio of 1:3. After 10 days of induction, the medium was replaced with NSC maintenance medium (N2B27 medium supplemented with 200 nM Hh-Ag 1.5, 3 μM CHIR-99021 and 12 μM SB-431542). After 5 passages in neural maintenance medium, the medium was changed to NSC medium (N2B27 supplemented with 20 ng/mL EGF and 20 ng/mL FGF2). The derived cells were subjected to the following assays.

### Immunofluorescence (IF) assay

An IF assay was performed based on our previous method 16. Briefly, undifferentiated H9 cells and NSCs derived from H9 cells were maintained on Matrigel-coated coverslips. Cells were incubated with mouse OCT4 (1:100, Santa Cruz Biotechnology), rabbit anti-PAX6 (1:100, Proteintech), rabbit anti-NESTIN (1:200, Sigma), mouse anti-GFAP (1:500, Proteintech) and goat anti-SOX1 (1:100, Bio-techne). Alexa Fluor 594 anti-rabbit (1:500), Alexa Fluor 488 anti-mouse (1:500) and anti-goat (1:500) antibodies were used as secondary antibodies. Visualisation of IF results was performed using a Leica SP8 confocal microscope.

### Cleavage Under Targets and Tagmentation (CUT&Tag) assay

The CUT&Tag assay was established according to a previous publication ^16^. Briefly, H9 cells and H9-derived spinal cord NSCs were subjected to the CUT&Tag assay as previously described^44^. The CUT&Tag assay was performed using anti-BAF45D antibodies. The enzyme pA-Tn5 transposase was used to precisely bind the DNA sequence near the BAF45D protein under antibody control and form factor targeted tagging. The DNA sequence is tagged at both ends with adapters.

Clustering of samples was performed using a cBot cluster generation system by TruSeq PE Cluster Kit v3-cBot-HS (Illumina). Library samples were sequenced on an Illumina NovaSeq platform (Novogene, Beijing, China) and paired-end reads were generated for 150 bp. FastQC (V0.11.5) was used to perform basic statistics on the quality of the raw reads. The read sequences in FASTQ format were then pre-processed using Trimmomatic software.

The reference genome index was created using BWA v0.7.12 (Burrows Wheeler Aligner), and clean reads were aligned to the reference genome using BWA mem v0.7.12. All peak calling was performed using MACS2. For peak calling simulations per input read, aligned and deduplicated BAM files were used without additional filtering. A peak annotator was used to identify the nearest TSS of each peak, and the distance distribution between peaks and TSS is shown. In addition, the distribution of peak summits on different functional regions, such as the 5’ UTR, CDS, and 3’UTR, was determined. Peak-related genes were confirmed by Peak Annotator, and then GO enrichment analysis was performed to identify the functional enrichment results. Visualisation of custom sequencing data together with conservation data was obtained using the UCSC Genome Browser (https://genome-asia.ucsc.edu/).

### Supplementary Information

Supplementary Information.

## Data Availability

The CUT&Tag data had been uploaded to GEO database with a linkage https://www.ncbi.nlm.nih.gov/geo/query/acc.cgi?acc=GSE190877, which can be accessed by others. The image data named IF data upload were uploaded as a related ZIP file if applicable.

## Abbreviations

NSC: Neural stem cells
BAF: BRG1/BRM-associated factor
PG: Paralogous groups
PRC: Polycomb repressive complexes
ARP: Actin-related protein
CUT&Tag: Cleavage Under Targets and Tagmentation
esBAF: Embryonic stem cell-specific BAF complex
npBAF: Neural progenitor BAF complex
nBAF: Neuronal BAF complex
NMP: Neural mesodermal progenitors
RFX: Regulatory factor X
