# Supplementary material for: BAF45D-binding to HOX genes was differentially targeted in H9-derived spinal cord neural stem cells
Source: Sci Rep. 2024 Jan 2;14:29. doi: 10.1038/s41598-023-50939-y (PMC10761701; doi:10.1038/s41598-023-50939-y)
Supplement: Supplementary file 1 — Supplementary Information. [file 41598_2023_50939_MOESM1_ESM.pdf]

Supplementary Information

BAF45D-binding to HOX genes was differentially targeted in H9-derived spinal cord neural stem cells

Chang Liu[1], Yuxin Xie[2], Xueying Chen[2], Lihua Liu[3], Chao Liu[2*] and Zongsheng Yin[1*]

1. Department of Orthopedics, The First Affiliated Hospital, Anhui Medical University, Hefei 230032, Anhui, China

2. **Department of Histology and Embryology, Institute of Stem Cell and Tissue Engineering,** School of Basic Medical Sciences, Anhui Medical University, Hefei, Anhui 230032, China

3. Institute of Clinical Pharmacology, Anhui Medical University, Hefei, Anhui 230032, China

Methods

IF assay

Undifferentiated H9 cells and H9-derived NSCs were maintained on Matrigel-coated coverslips. Cells were incubated with mouse anti-PAX6 (1:100, Abcam), anti-HOXC9 (1:100, Abcam), anti-HOXB1(1:25 ,Abnova), rabbit anti-HOXB13(1:200 ,Poteintech), rabbit anti-NESTIN (1:200, Sigma) and rabbit anti-BAF45D (1:100, Proteintech) antibodies. Alexa Fluor 594 anti-rabbit (1:500) and Alexa Fluor 488 anti-mouse (1:500) antibodies were used as secondary antibodies. Visualisation of IF results was performed using a Nikon Eclipse 80i fluorescence microscope.

Over expression of GFP-tagged BAF45D in P19 cells and RA induction

P19 cell was cultured and transfected with plasmids as previously described (Liu, Zhang et al. 2015). Briefly, the cells were transfected with plasmids expressing GFP and GFP-tagged BAF45D by using Lipofectamine 2000 (Invitrogen, Carlsbad, CA).

Then the transfected P19 cells were exposured by RA and subjected to the following IB assay using the indicated antibodies.

Immunoblotting (IB) assays

The lysates of cells were subjected to IB assay according to a previous protocol(Chen 2022) . Proteins were separated by SDS–PAGE and transferred onto polyvinylidene difluoride membranes (Millipore, Temecula, CA, USA). Then, the proteins were detected using the indicated antibodies. The following primary antibodies were used: mouse anti-GFP(1:10000, Proteintech),mouse anti-HOXC9 (1:1000, Abcam) and rabbit anti-GAPDH

(1:5000, Proteintech).

Gene ontology (GO) analysis

The GO assay were performed using topGO software, a package for Bioconductor (version 2.13) (https://bioconductor.org/packages/2.13/bioc/html/topGO.html). The GO raw data include the data of both undifferentiated H9 cells and H9-derived spinal cord NSCs, as well as the data of the GO enrichment analysis of differential genes between the two cell types. The descriptions with the "neural tube" were selected first. Then the corrected P values associated with the descriptions were calculated. The top five "Log10pValues" of the selections were plotted in the bar graphs.

Function analysis of BAF45D bound regions

To address the function of BAF45D bound regions, we used publicly available Chip-seq data sets (http://cistrome.org/db/#/) of both H9 cells and H9-derived neural progenitor cells published in a previous paper (Ziller, Edri et al. 2015). The data sets were analysed using the UCSC genome browser linked to the website. The levels of H3K27ME3 and H3K27AC were plotted across the *DPF2* locus.

Results

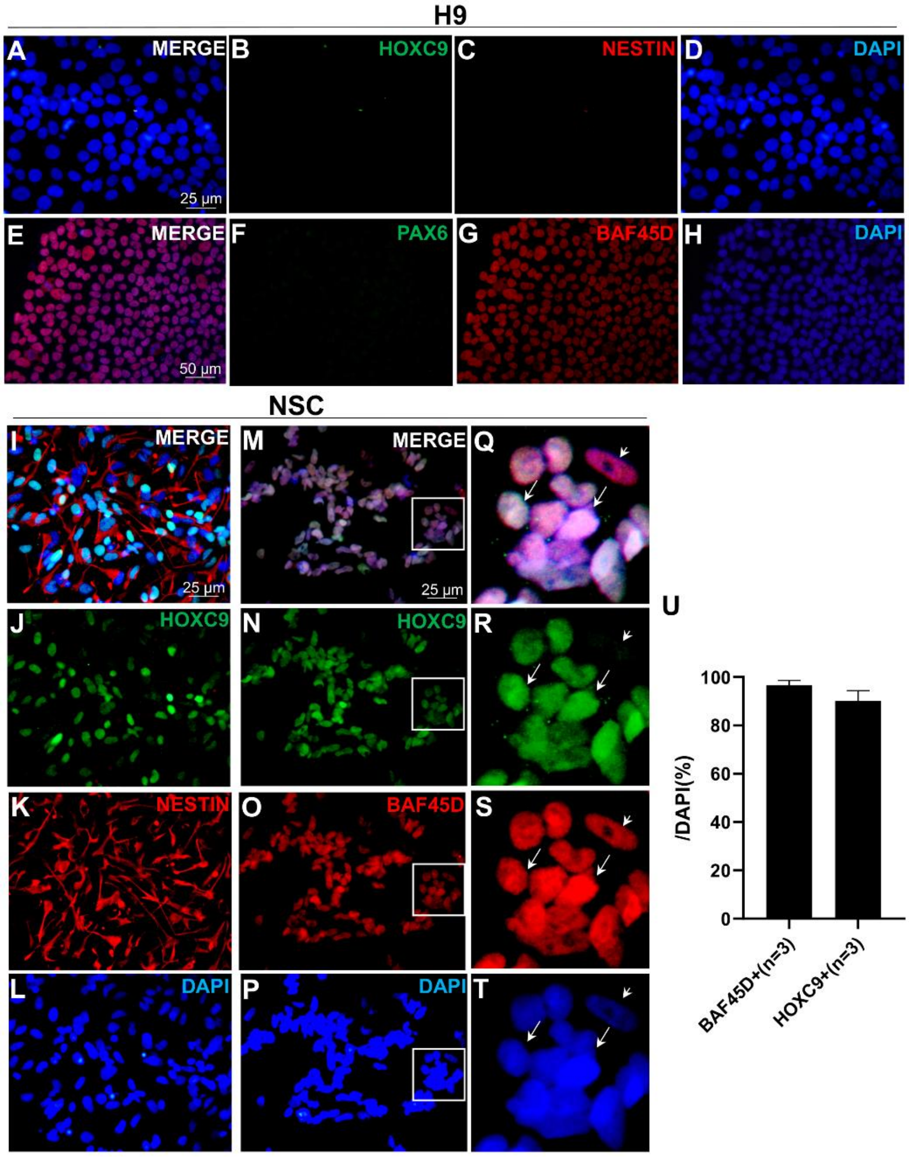

Figure S1. BAF45D colocalised with HOXC9 in H9-derived spinal cord NSCs.

A-H, IF assays were performed for the expression of HOXC9 (green)、NESTIN (red)、PAX6 and BAF45D in H9 cells ***I-L***, IF assay was performed for the expression of HOXC9 (green) and NESTIN (red) in the H9-derived spinal cord NSCs. *M-T*, IF assay was performed for the expression of HOXC9 (green) and BAF45D (red) in the H9-derived spinal cord NSCs. Q, R, S and T are the insets in M, N, O and P, respectively. Arrows indicate colocalisation of HOXC9 and BAF45D. Arrowheads indicate expression of BAF45D only. Nuclei were counterstained with DAPI (blue). Scale bar=25μm (A-D,I-P). Scale bar=50μm (E-H). U, The statistical analysis for the cells positive for HOXC9 and BAF45D.

A Homer known motif enrichment results (NSC1)

Total Target Sequences = 34181, Total Background Sequences = 31669

Rank	Motif	Name	P-value	log P-pvalue	q-value (Benjamini)	# Target Sequences with Motif	% of Targets Sequences with Motif
1	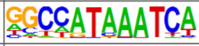	Hoxc9(Homeobox)/Ainv15-Hoxc9-ChIP-Seq(GSE21812)/Homer	1e-1075	-2.476e+03	0.0000	8200.0	23.99%
2	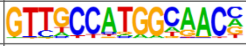	Rfx2(HTH)/LoVo-RFX2-ChIP-Seq(GSE49402)/Homer	1e-859	-1.979e+03	0.0000	2413.0	7.06%
3	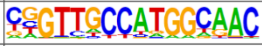	RFX(HTH)/K562-RFX3-ChIP-Seq(SRA012198)/Homer	1e-813	-1.873e+03	0.0000	2281.0	6.67%
4	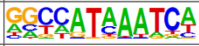	HOXA9(Homeobox)/HSC-Hoxa9-ChIP-Seq(GSE33509)/Homer	1e-809	-1.864e+03	0.0000	8919.0	26.09%
5	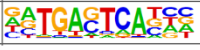	Fosl2(bZIP)/3T3L1-Fosl2-ChIP-Seq(GSE56872)/Homer	1e-752	-1.732e+03	0.0000	4391.0	12.85%

B Homer known motif enrichment results (NSC2)

Total Target Sequences = 27790, Total Background Sequences = 25718

Rank	Motif	Name	P-value	log P-pvalue	q-value (Benjamini)	# Target Sequences with Motif	% of Targets Sequences with Motif
1	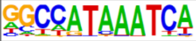	Hoxc9(Homeobox)/Ainv15-Hoxc9-ChIP-Seq(GSE21812)/Homer	1e-849	-1.957e+03	0.0000	6489.0	23.35%
2	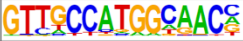	Rfx2(HTH)/LoVo-RFX2-ChIP-Seq(GSE49402)/Homer	1e-722	-1.663e+03	0.0000	2005.0	7.21%
3	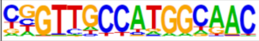	RFX(HTH)/K562-RFX3-ChIP-Seq(SRA012198)/Homer	1e-702	-1.618e+03	0.0000	1907.0	6.86%
4	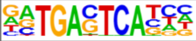	Jun-AP1(bZIP)/K562-cJun-ChIP-Seq(GSE31477)/Homer	1e-610	-1.405e+03	0.0000	2834.0	10.20%
5	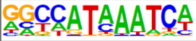	HOXA9(Homeobox)/HSC-Hoxa9-ChIP-Seq(GSE33509)/Homer	1e-591	-1.363e+03	0.0000	7003.0	25.20%

C Homer known motif enrichment results (ESC1)

Total Target Sequences = 4958, Total Background Sequences = 36619

Rank	Motif	Name	P-value	log P-pvalue	q-value (Benjamini)	# Target Sequences with Motif	% of Targets Sequences with Motif
1	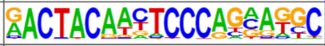	Ronin(THAP)/ES-Thap11-ChIP-Seq(GSE51522)/Homer	1e-97	-2.246e+02	0.0000	175.0	3.53%
2	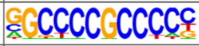	Sp1(Zf)/Promoter/Homer	1e-94	-2.183e+02	0.0000	802.0	16.19%
3	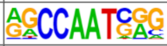	NFY(CCAAT)/Promoter/Homer	1e-62	-1.449e+02	0.0000	1175.0	23.71%
4	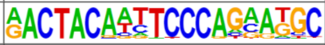	GFY-Staf(?,Zf)/Promoter/Homer	1e-56	-1.297e+02	0.0000	207.0	4.18%
5	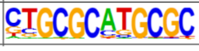	NRF1(NRF)/MCF7-NRF1-ChIP-Seq(Unpublished)/Homer	1e-53	-1.240e+02	0.0000	294.0	5.93%

D Homer known motif enrichment results (ESC2)

Total Target Sequences = 14008, Total Background Sequences = 32110

Rank	Motif	Name	P-value	log P-pvalue	q-value (Benjamini)	# Target Sequences with Motif	% of Targets Sequences with Motif
1	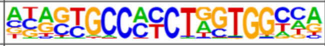	CTCF(Zf)/CD4+-CTCF-ChIP-Seq(Barski_et_al.)/Homer	1e-518	-1.194e+03	0.0000	1695.0	12.10%
2	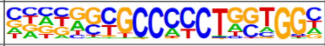	BORIS(Zf)/K562-CTCFL-ChIP-Seq(GSE32465)/Homer	1e-355	-8.188e+02	0.0000	2197.0	15.68%
3	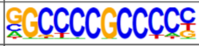	Sp1(Zf)/Promoter/Homer	1e-303	-6.994e+02	0.0000	2898.0	20.68%
4	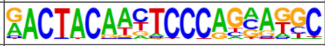	Ronin(THAP)/ES-Thap11-ChIP-Seq(GSE51522)/Homer	1e-281	-6.472e+02	0.0000	542.0	3.87%
5	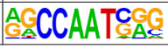	NFY(CCAAT)/Promoter/Homer	1e-265	-6.108e+02	0.0000	3510.0	25.05%

.

Figure S2. Homer known motif enrichment results of H9 cells and H9-derived spinal cord NSCs.

A* and *B, The top five motifs of BAF45D bound genes identified in the H9-derived spinal cord NSCs. ***C* and *D***, The top five motifs of BAF45D bound genes identified in the H9 cells.

A

Homer *de novo* Motif Results (NSC1/)

Known Motif Enrichment Results
Gene Ontology Enrichment Results
If Homer is having trouble matching a motif to a known motif, try copy/pasting the matrix file into STAMP
More information on motif finding results: HOMER | Description of Results | Tips
Total target sequences = 34181
Total background sequences = 31655
* - possible false positive

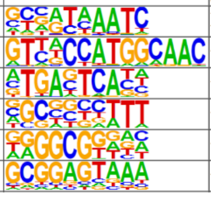

Rank	Motif	P-value	log P-pvalue	% of Targets	% of Background	STD(Bg STD)	Best Match/Details	Motif File
1		1e-1019	-2.348e+03	35.73%	19.81%	130.2bp (149.2bp)	Hoxc9/MA0485.1/Jaspar(0.906) More Information | Similar Motifs Found	motif file (matrix)
2		1e-893	-2.058e+03	6.93%	1.33%	107.3bp (168.6bp)	Rfx1/MA0509.1/Jaspar(0.963) More Information | Similar Motifs Found	motif file (matrix)
3		1e-775	-1.786e+03	11.82%	4.05%	103.9bp (144.7bp)	BATF(bZIP)/Th17-BATF-ChIP-Seq(GSE39756)/Homer(0.990) More Information | Similar Motifs Found	motif file (matrix)
4		1e-561	-1.293e+03	65.32%	51.73%	141.4bp (158.4bp)	DOF5.7/MA0984.1/Jaspar(0.709) More Information | Similar Motifs Found	motif file (matrix)
5		1e-506	-1.165e+03	21.95%	12.55%	119.4bp (189.1bp)	btd/MA0443.1/Jaspar(0.901) More Information | Similar Motifs Found	motif file (matrix)
6		1e-506	-1.165e+03	64.77%	51.87%	137.9bp (165.1bp)	CHA4/MA0283.1/Jaspar(0.773) More Information | Similar Motifs Found	motif file (matrix)

B

Homer *de novo* Motif Results (NSC2/)

Known Motif Enrichment Results
Gene Ontology Enrichment Results
If Homer is having trouble matching a motif to a known motif, try copy/pasting the matrix file into STAMP
More information on motif finding results: HOMER | Description of Results | Tips
Total target sequences = 27799
Total background sequences = 25715
* - possible false positive

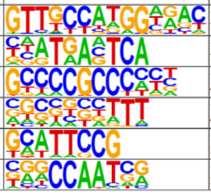

Rank	Motif	P-value	log P-pvalue	% of Targets	% of Background	STD(Bg STD)	Best Match/Details	Motif File
1		1e-797	-1.836e+03	7.30%	1.34%	107.3bp (156.2bp)	Rfx1/MA0509.1/Jaspar(0.975) More Information | Similar Motifs Found	motif file (matrix)
2		1e-759	-1.749e+03	22.90%	10.57%	117.0bp (147.2bp)	FOS/MA0476.1/Jaspar(0.938) More Information | Similar Motifs Found	motif file (matrix)
3		1e-496	-1.143e+03	12.95%	5.40%	111.3bp (185.4bp)	Sp1(Zf)/Promoter/Homer(0.959) More Information | Similar Motifs Found	motif file (matrix)
4		1e-403	-9.298e+02	51.46%	38.71%	140.8bp (158.9bp)	DOF5.7/MA0984.1/Jaspar(0.774) More Information | Similar Motifs Found	motif file (matrix)
5		1e-387	-8.934e+02	70.43%	58.20%	141.0bp (164.8bp)	LYS14/MA0325.1/Jaspar(0.868) More Information | Similar Motifs Found	motif file (matrix)
6		1e-355	-8.194e+02	10.47%	4.60%	111.3bp (165.5bp)	NFY(CCAAT)/Promoter/Homer(0.947) More Information | Similar Motifs Found	motif file (matrix)

C

Homer *de novo* Motif Results (ESC1/)

Known Motif Enrichment Results
Gene Ontology Enrichment Results
If Homer is having trouble matching a motif to a known motif, try copy/pasting the matrix file into STAMP
More information on motif finding results: HOMER | Description of Results | Tips
Total target sequences = 4953
Total background sequences = 35967
* - possible false positive

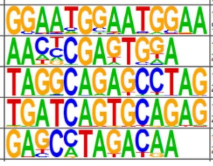

Rank	Motif	P-value	log P-pvalue	% of Targets	% of Background	STD(Bg STD)	Best Match/Details	Motif File
1		1e-599	-1.381e+03	8.23%	0.11%	124.3bp (321.6bp)	HSF1/HSF1_H2O2Lo/11-HSF1(Harbison)/Yeast(0.678) More Information | Similar Motifs Found	motif file (matrix)
2		1e-274	-6.313e+02	3.09%	0.02%	125.6bp (3.3bp)	tin/MA0247.2/Jaspar(0.657) More Information | Similar Motifs Found	motif file (matrix)
3		1e-259	-5.979e+02	2.12%	0.00%	131.9bp (94.0bp)	MOT3/MA0340.1/Jaspar(0.567) More Information | Similar Motifs Found	motif file (matrix)
4		1e-219	-5.052e+02	2.20%	0.01%	136.4bp (215.5bp)	GATA15(C2C2gata)/col-GATA15-DAP-Seq(GSE60143)/Homer(0.597) More Information | Similar Motifs Found	motif file (matrix)
5		1e-216	-4.989e+02	1.82%	0.00%	130.0bp (0.0bp)	SMAD3/MA0795.1/Jaspar(0.628) More Information | Similar Motifs Found	motif file (matrix)

D

Homer *de novo* Motif Results (ESC2/)

Known Motif Enrichment Results
Gene Ontology Enrichment Results
If Homer is having trouble matching a motif to a known motif, try copy/pasting the matrix file into STAMP
More information on motif finding results: HOMER | Description of Results | Tips
Total target sequences = 14017
Total background sequences = 32095
* - possible false positive

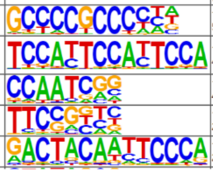

Rank	Motif	P-value	log P-pvalue	% of Targets	% of Background	STD(Bg STD)	Best Match/Details	Motif File
1		1e-521	-1.202e+03	19.11%	6.67%	122.2bp (197.0bp)	Sp1(Zf)/Promoter/Homer(0.965) More Information | Similar Motifs Found	motif file (matrix)
2		1e-457	-1.055e+03	2.81%	0.08%	122.8bp (243.2bp)	MOD(RRM)/Drosophila_melanogaster-RNCMPT00140-PBM/HughesRNA(0.655) More Information | Similar Motifs Found	motif file (matrix)
3		1e-401	-9.244e+02	32.91%	17.83%	122.6bp (166.1bp)	NFY(CCAAT)/Promoter/Homer(0.925) More Information | Similar Motifs Found	motif file (matrix)
4		1e-269	-6.216e+02	30.14%	17.93%	136.0bp (183.9bp)	Elk1(ETS)/Hela-Elk1-ChIP-Seq(GSE31477)/Homer(0.852) More Information | Similar Motifs Found	motif file (matrix)
5		1e-251	-5.799e+02	3.83%	0.57%	103.6bp (191.9bp)	GFY(?)/Promoter/Homer(0.942) More Information | Similar Motifs Found	motif file (matrix)

Figure S3. Homer *de novo* motif results of H9 cells and H9-derived spinal cord NSCs.
A* and *B, The top five de novo motifs for BAF45D identified in the H9-derived spinal cord NSCs. ***C* and *D***, The top five de novo motifs for BAF45D identified in the H9 cells.

A

GO peak NSC vs ESC

GO_accession	Description	Term_type	Corrected_pValue	-Log10pValue
GO:0021915	neural tube development	biological_process	2.58E-05	4.59E+00
GO:0001841	neural tube formation	biological_process	0.0033971	2.47E+00
GO:0014020	primary neural tube formation	biological_process	0.010837	1.97E+00
GO:0001843	neural tube closure	biological_process	0.012265	1.91E+00
GO:0060831	smoothened signaling pathway involved in dorsal/ventral neural tube patterning	biological_process	0.021376	1.67E+00

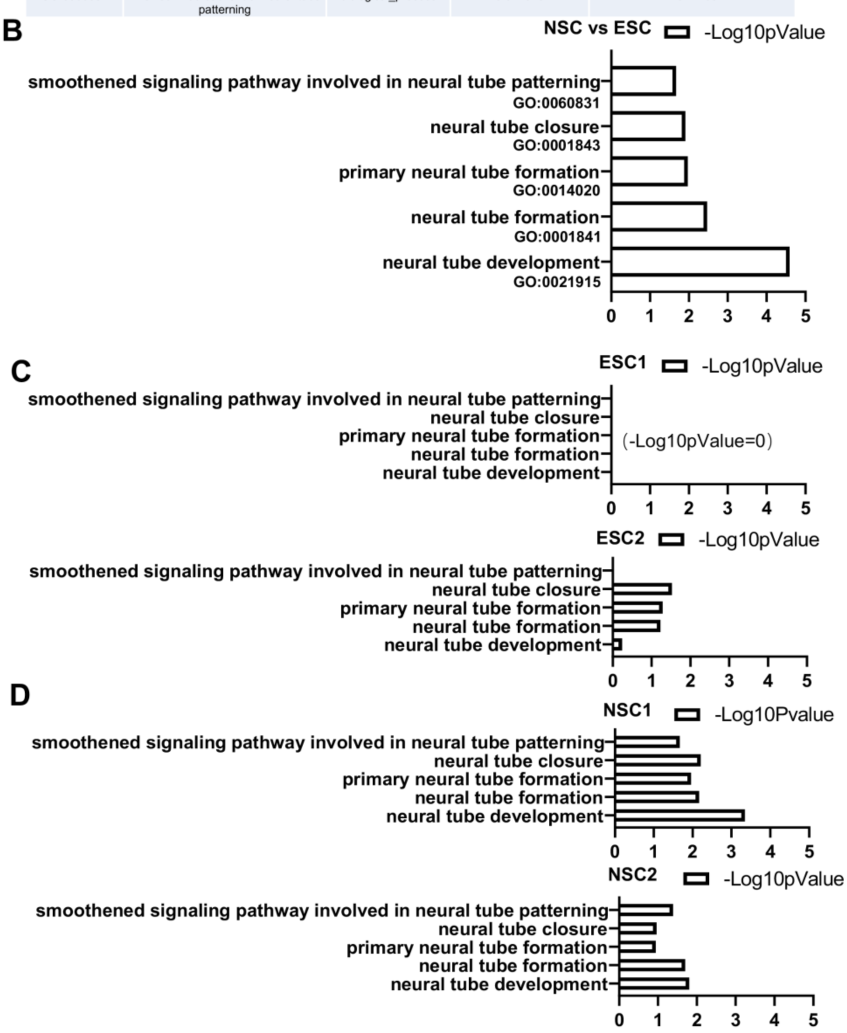

Figure S4. Gene Ontology analysis of BAF45D binding events for some significantly enriched biological processes associated with neural tube development.

A, For the selection of GO terms,the descriptions with "neural tube" were selected first. Then "-Log10pValues" were calculated based on the corrected P-values associated with the descriptions. The top five "-Log10pValues" were plotted in the bar graphs. ***B-D***, Functional annotation based on Gene Ontology (GO) categorization in the descriptions in the panel A. The horizontal bar represents the enrichment of the genes associated with biological processes identified in H9 cells vs. NSCs (B), and the enrichment of the same genes in H9 cells (C) and the NSCs (D), respectively.

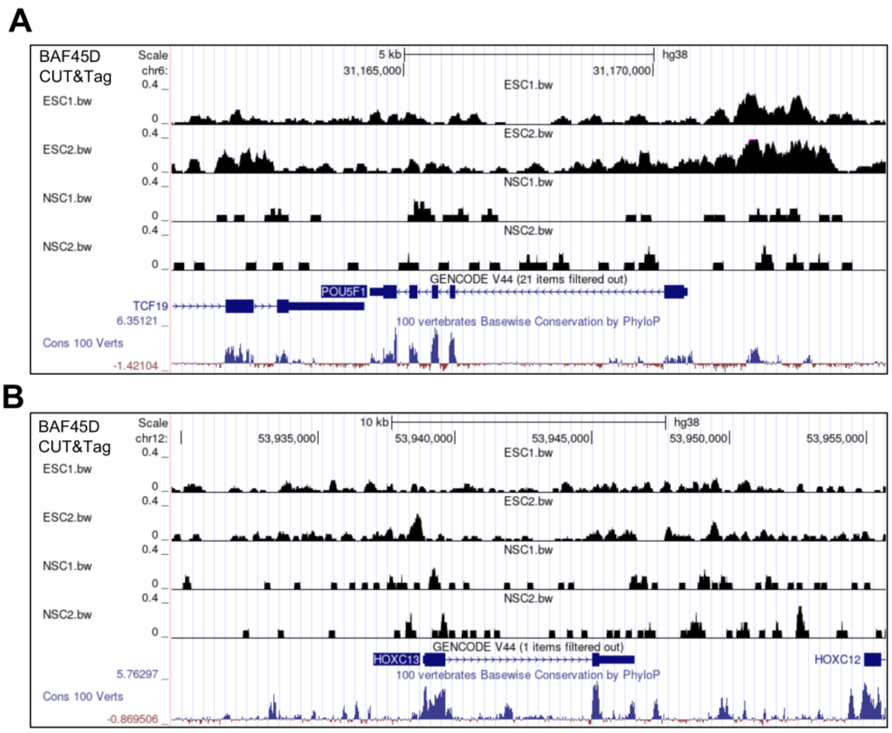

Figure S5. BAF45D binding to POU5F1 and HOXC13 genes was less enriched in NSCs chromatin compared to ESCs chromatin.

A, Chromatin accessibility of BAF45D-bound POU5F1, a stem cell marker, was decreased in NSCs compared to H9 cells. ***B***, Chromatin accessibility of BAF45D-bound HOXC13, a posterior spinal cord marker, was decreased in NSCs compared to H9 cells.

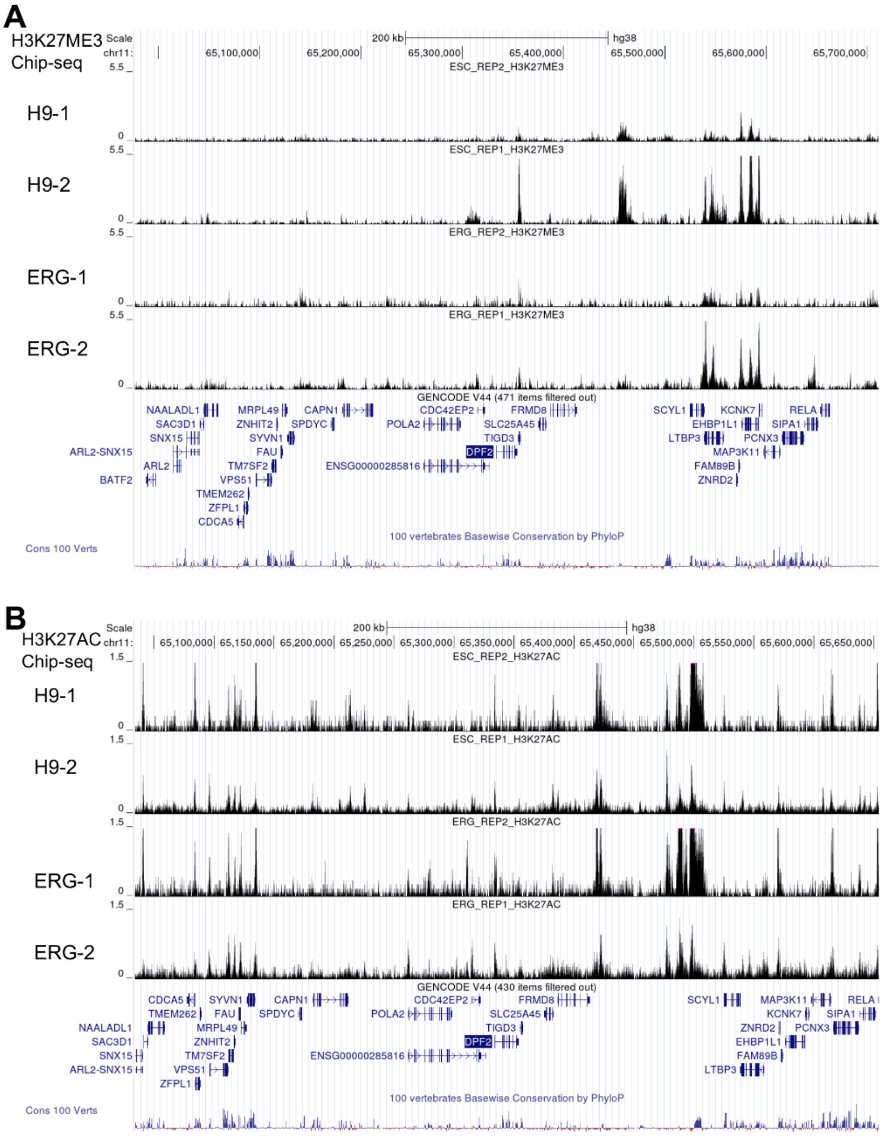

Figure S6. ChIP-seq enrichment profiles of H3K27ME3 and H3K27AC histone marks in across genomic regions centered at BAF45D peak summits.

A, ChIP-seq enrichment profiles of H3K27ME3 histone marks in across genomic regions centered at BAF45D peak summits. ***B***, ChIP-seq enrichment profiles of H3K27AC histone marks in across genomic regions centered at BAF45D peak summits.

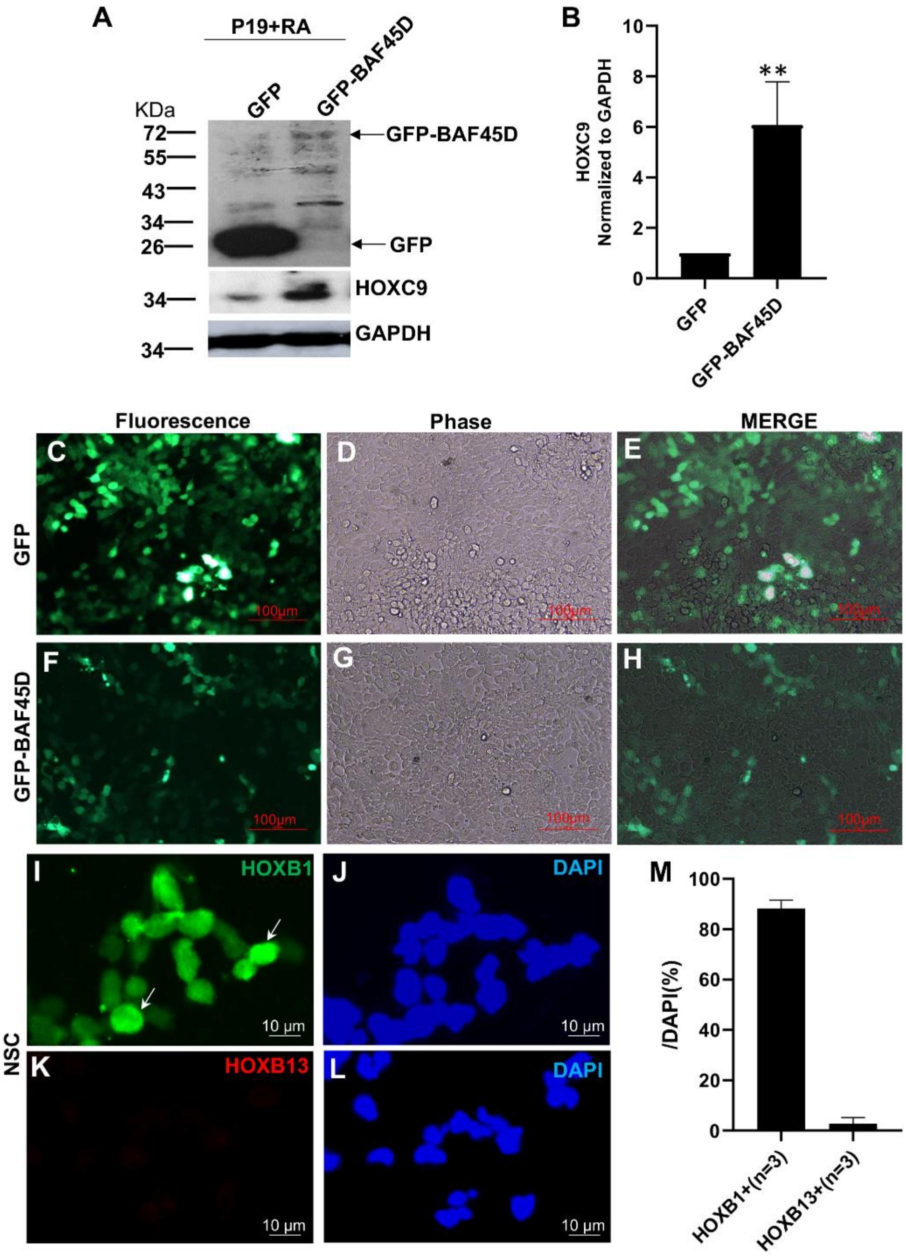

Figure S7. BAF45D increased HOXC9 expression in RA treated P19 cells and H9-derived neural stem cells expressed HOXB1 but few or no HOXB13.

A, Forced expression of BAF45D in P19 cells and the effect of BAF45D on HOXC9 expression in the transfected P19 cells treated by RA. ***B***, Statistical analysis indicated that the expression of HOXC9 was significantly increased by GFP-BAF45D as compared to the GFP. ***C-H,*** Fluorescence (C and F) and phase(D and G) micrographs of the P19 cells with expression of GFP(C and E) and GFP-BAF45D (F-H). ***I-L***, H9-derived neural stem cells expressed robust HOXB1(green, I-J) but few or no HOXB13 (red, K-L). Arrows indicate the robust expression of HOXB1. Scale bar=10μm. ***M，*** The statistical analysis for the cells positive for HOXB1 and HOXB13.

Reference

Chen, X., et al (2022). "BAF45D regulates spinal cord neural stem/progenitor cell fate through the SMAD-PAX6 axis." Genes & Diseases.

Liu, C., D. J. Zhang, Y. X. Shen, X. F. Tao, L. H. Liu, Y. W. Zhong and S. Y. Fang (2015). "DPF2 regulates OCT4 protein level and nuclear distribution." Biochimica Et Biophysica Acta-Molecular Cell Research **1853**(12): 3279-3293.

Ziller, M. J., R. Edri, Y. Yaffe, J. Donaghey, R. Pop, W. Mallard, R. Issner, C. A. Gifford, A. Goren, J. Xing, H. Gu, D. Cachiarelli, A. Tsankov, C. Epstein, J. R. Rinn, T. S. Mikkelsen, O. Kohlbacher, A. Gnirke, B. E. Bernstein, Y. Elkabetz and A. Meissner (2015). "Dissecting neural differentiation regulatory networks through epigenetic footprinting." Nature **518**(7539): 355-359.
